# The clinical characteristics and outcomes of carotid body tumors in Chinese patients: A STROBE-compliant observational study

**DOI:** 10.1097/MD.0000000000018824

**Published:** 2020-01-17

**Authors:** Yangjing Chen, Yanzi Li, Jianlin Liu, Lin Yang

**Affiliations:** a Department of Otolaryngology; b Department of Medical Administration; c Department of Vascular Surgery, First Affiliated Hospital of Xi’an Jiaotong University, Xi’an, China.

**Keywords:** carotid body tumor, complications, embolization, outcomes, population

## Abstract

This study aims to investigate the clinical characteristics and outcomes of carotid body tumors in Chinese patients in the last decade. A systematic search was conducted without limits and included studies published between January 2006 and December 2016 according to PubMed, the Chinese Science Citation Database, the China Science Periodical Database and the China National Knowledge Infrastructure. Relevant synonyms for the search terms “paraganglioma” and “carotid body tumor” were applied, and the clinical data were evaluated and analyzed. There were 1810 cases of CBTs reported in the last decade, of which females accounted for 60.22%, and the mean age was 40.60 years, with most cases being sporadic (98.51%). Surgical resection was performed in 1791 cases: vessel repair occurred in 38.88% of the cases, carotid ligation occurred in 1.42% of the cases, and 1.05% of the patients refused treatment. Some patients underwent selective embolization, and the results showed that embolization could decrease procedure time and blood loss (*P* < .01). Stroke and death occurred in 1.95% and 0.39% of patients, respectively. Malignant CBTs accounted for 4.30% of cases, and the metastatic sites involved were local metastasis (46.88%), lung (31.25%), bone (21.88%), liver (12.50%), and brain (9.38%). The overall survival rate was 98.87% 36 months after the procedure, and the survival rate of metastatic cases was 56.25% 6 months after recurrence; however, only 21.88% of metastatic cases received radiotherapy. The CBTs of Chinese patients showed some clinical features that were different from those of Western patients.

## 1 Introduction

Carotid body tumors (CBTs) are rare vascular tumors of the head and neck that originate from neural crest paraganglion cells.^[1,2]^ CBTs account for 65% of head and neck paragangliomas; ∼90% of CBTs are sporadic, and 10% are familial.^[3–5]^ Although most CBTs are usually benign, the metastatic rate is ∼5%^[6,7]^; however, malignancy can be determined by the presence of vascular invasion, perineural invasion, and metastasis.^[8]^ Early surgical excision has been recommended for CBTs to reduce the risk of perioperative complications and malignancy.^[9]^ Moreover, preoperative embolization has been found to be an effective and safe method for surgical resection that helps to diminish intraoperative bleeding and malignancy.^[10,11]^

Since CBTs are rare, population-level data represent an ideal way to evaluate and assess their clinical outcomes. Although a few CBT cases and associated clinical therapy choices in Chinese patients have been reported in the literature, no large-scale study of CBTs in Chinese patients has been published yet. Therefore, we conducted this study to assess the clinical outcomes and the current treatment progress of CBTs over a decade, from January 2006 to December 2016, in China.

## 2 Methods

### 2.1 Data collection

National published data and reports detailing Chinese CBT patients from January 2006 to December 2016 from PubMed, the Chinese Science Citation Database, the China Science Periodical Database, and the Chinese National Knowledge Infrastructure were evaluated. Relevant synonyms for the search terms “paraganglioma” and “carotid body tumor” were applied. Full manuscripts were reviewed when studies appeared appropriate.

### 2.2 Study procedure

For the purpose of this study, all patients with CBTs who were hospitalized electively and underwent surgical resection with or without secondary procedures were of interest. The inclusion criteria for the reports evaluated were as follows:

(1) the paper must include integrity data of the serial cases;
(2) at least 3 cases must be included in the paper; and
(3) if several papers were published by one institute, only the one with the largest number of serial cases was included.

This study was approved by the institutional review board of the First Affiliated Hospital of Xi’an Jiaotong University.

Only data from patients diagnosed with CBTs were included, and data from patients with vagal paragangliomas or other paraganglia tumors were excluded. All of the demographic data, baseline data, lesion characteristics, risk factors, associated diseases, and perioperative examination methods were collected. We analyzed the surgical procedures and perioperative complications as well as whether preoperative embolization occurred. The follow-up and mortality data were analyzed; moreover, the Elixhauser comorbidity variables were used to identify and adjust for comorbidities in this study cohort.^[12]^

### 2.3 Statistical analysis

SAS 9.1 software (SAS Institute, Cary, NC) was used for the analysis of the databases and all statistics. To test the difference between groups, we used Chi-square analysis for categorical variables and Student's *t* test and analysis of variance (ANOVA) for continuous variables, and we tested the significance of the difference between 2 independent proportions when the results were presented as percentages. All reported *P* values are 2-sided; *P* < .05 was considered significant.

## 3 Results

### 3.1 The demographic data of CBT patients

**Figure 1:**
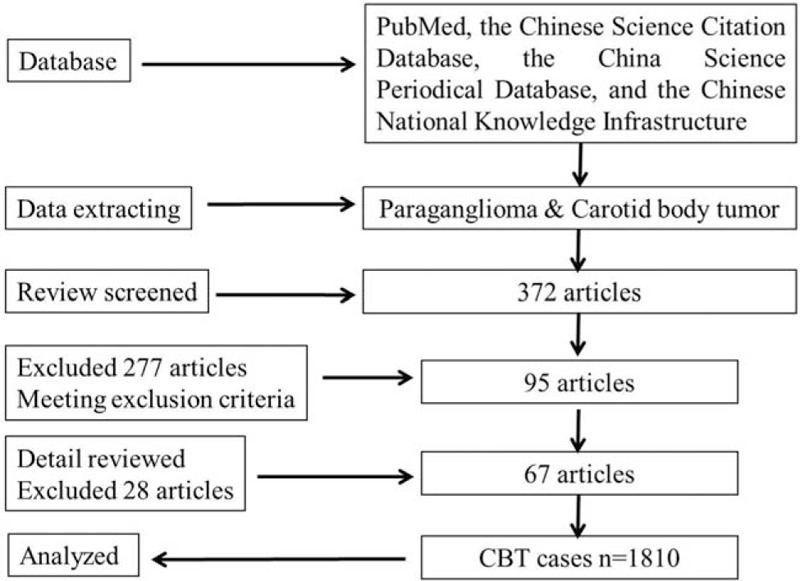
The flow chart of the study.

**Figure 2:**
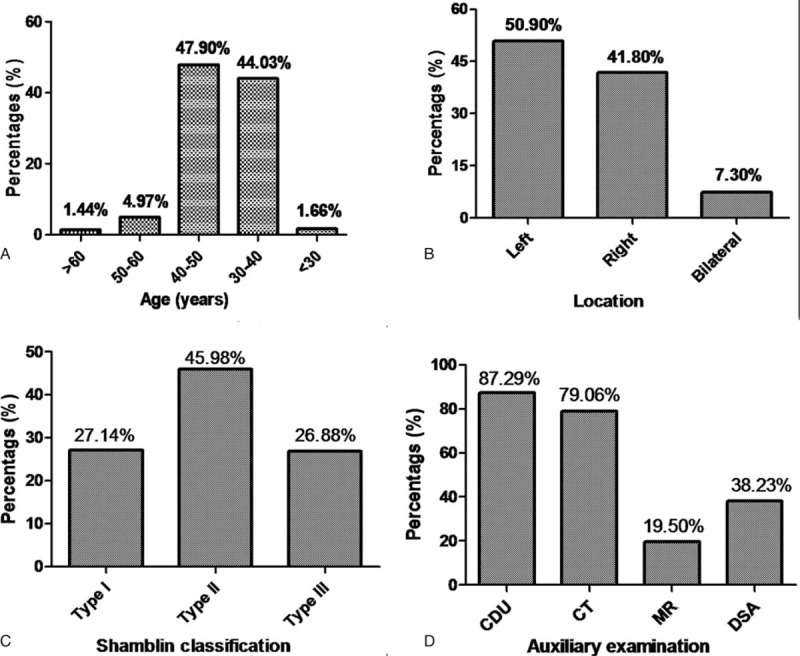
The demographic and baseline features of CBTs. (A) Age at onset; (B) location of tumors; (C) Shamblin classification; (D) auxiliary examination methods.

We identified 372 articles from the abovementioned databases (Fig. 1). Two reviewers independently screened the identified titles and abstracts, and 95 studies were ultimately retrieved for a detailed review. Finally, 67 retrospective reports were ultimately included. A total of 1810 patients were involved, of whom 720 were male, resulting in a male-to-female ratio of 1:1.51. Effective age information was included in 1607 cases (88.78%, Fig. 2A), and while this disease may occur at any age, the mean age was 40.60 years old (range: 7–77 years); the peak ages of onset were 30 to 40 years old (44.03%) and 40 to 50 years old (47.90%). Effective clinical course data were collected in 485 cases (26.80%), and the mean duration of history was 44.8 months (range: 1 week to 30 years).

### 3.2 The baseline features of CBTs

Most cases were unilateral (Fig. 2B), with 921 cases occurring at the left carotid bifurcation (50.90%) and 132 cases demonstrating bilateral tumors (7.30%). A familial history was confirmed in 27 cases (1.49%), with the other 1783 cases being sporadic CBTs (98.51%). In addition, 205 high-altitude resident patients were diagnosed with CBTs (11.33%). According to the Shamblin classification of CBTs listed in Figure 2C, 527 tumors were Shamblin type I, 893 were type II, and 522 were type III, resulting in a Shamblin ratio of ∼1:1.7:1. The mean tumor diameter was 4.39 cm (range: 0.8–13.5 cm) in 1611 cases (89.01%). The most common auxiliary examination used for the diagnosis of CBTs was color Doppler ultrasound (1580 cases, Fig. 2D). Additional examination methods included computed tomography (CT, 1431 cases), magnetic resonance imaging (MRI, 353 cases), and digital subtraction angiography (DSA, 692 cases).

### 3.3 The clinical manifestations of CBTs

**Figure 3:**
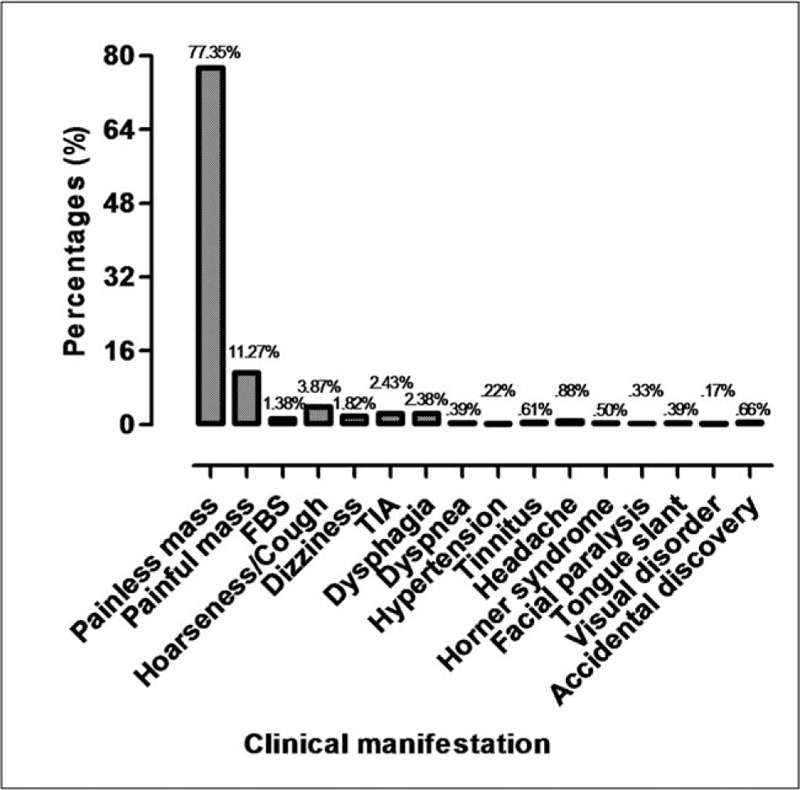
The clinical manifestations of CBTs.

In this study, the most common clinical presentation of CBTs was a painless neck mass (1400 cases, Fig. 3), which was reported in 204 patients. The following symptoms were identified: the sensation of a foreign body in the neck (25 cases), hoarseness and cough (70 cases), dizziness (33 cases), transient ischemic attack (44 cases), and dysphagia (43 cases). Rare symptoms included the following: dyspnea (7 cases), hypertension (4 cases), tinnitus (11 cases), headache (16 cases), Horner syndrome (9 cases), facial paralysis (6 cases), tongue slant (7 cases), and visual disorders (3 cases). In addition, CBTs were accidentally discovered in some patients when they underwent neck surgery for other reasons (12 cases).

### 3.4 The outcomes of surgical resection

There were 1791 patients (1826 tumors) who underwent surgical resection (98.95%, Table 1) and 19 patients who refused therapy (1.05%). The patients with bilateral CBTs underwent simultaneous resections in 35 cases (26.52%) and staged resections in 97 cases (73.48%). Complete tumor resection was performed in 1090 cases (59.69%), while 356 patients underwent external carotid artery partial resection, and 121 patients underwent internal carotid artery partial resection. Vessel replacement was performed in 233 cases (12.76%), and a carotid shunt was used for 69 cases (3.78%). Replacement materials included autogenous veins (68.67%), grafts (19.31%), and the external carotid artery (12.02%). Carotid artery ligation was performed in 26 great tumor cases (1.42%).

**Table 1:**
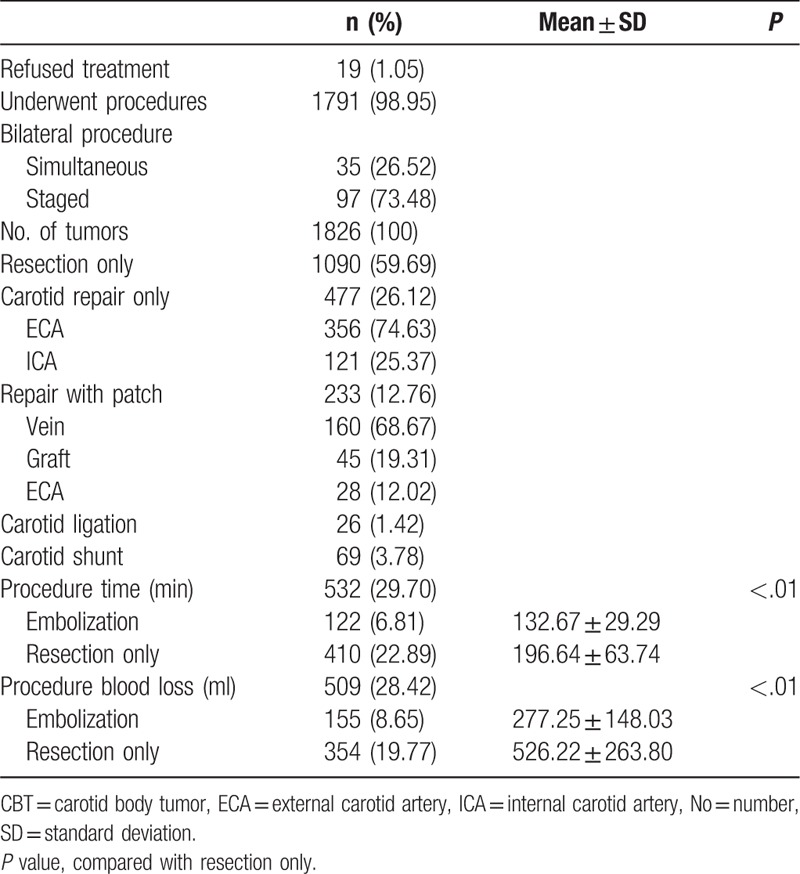
The surgical procedure data of CBT patients.

### 3.5 Outcome of resection with or without preoperative embolization

We collected relatively complete comparison data of intraoperative blood loss in 155 patients who underwent preoperative embolization and 354 patients who did not. Intraoperative blood loss was significantly decreased in the preoperative embolization group (277.25 ± 148.03 mL vs 526.22 ± 263.80 mL, *P* = .006). Complete procedure time data were collected in 122 patients who underwent embolization and 410 patients who did not. The procedure time was significantly shorter in the embolized group than in the nonembolized group (132.67 ± 29.29 min vs 196.64 ± 62.74 mL, *P* = .003).

### 3.6 The complications of surgical resection

**Figure 4:**
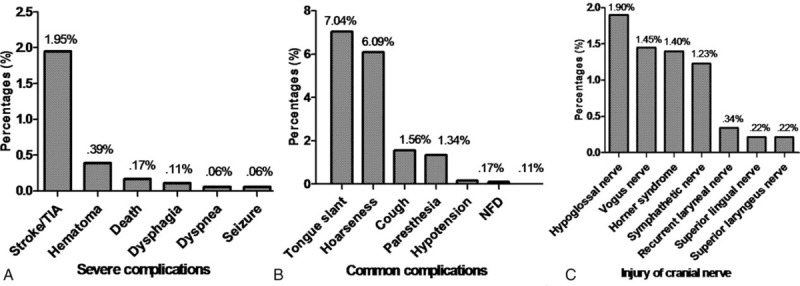
The incidence rate of complications after surgical procedures. (A) The incidence rate of severe complications; (B) the most common complications; (C) the incidence rate of injury to cranial nerves.

The complications of surgical resection are shown in Figure 4. Severe complications included stroke/transient ischemic attack (TIA) (35 cases, Fig. 4A), hematoma at the incision (7 cases), death (3 cases), dysphasia (2 cases), seizure (1 case), and dyspnea (1 case). Common complications included tongue slant (126 cases, Fig. 4B), hoarseness (109 cases), cough (28 cases), paresthesia of the neck (24 cases), hypotension (3 cases), and nasolabial fold disappearance (2 cases). There were no data related to the complications of incision infection and deep vein thrombosis. Complications due to injuries of the cranial nerves after operations (Fig. 4C) included Horner syndrome (25 cases) as well as injuries to the hypoglossal nerve (34 cases), vagus nerve (26 cases), sympathetic nerves (22 cases), recurrent laryngeal nerve (6 cases), superior lingual nerve (4 cases), and superior laryngeal nerve (4 cases).

### 3.7 Pathological results and follow-up

Based upon the pathological examination of all specimens, 77 cases showed characteristics of malignant tumors, local lymph node metastases were confirmed in 61 cases (79.22%), and the other 16 cases were confirmed based upon clinical characteristics (20.78%). There were 1484 cases with completed follow-up data (82.86%) with a mean follow-up time of 36.31 ± 20.04 months (range 1 month to 35 years), the overall survival rate during follow-up was 98.87% by Kaplan–Meier method.

### 3.8 The metastasis of CBTs

**Figure 5:**
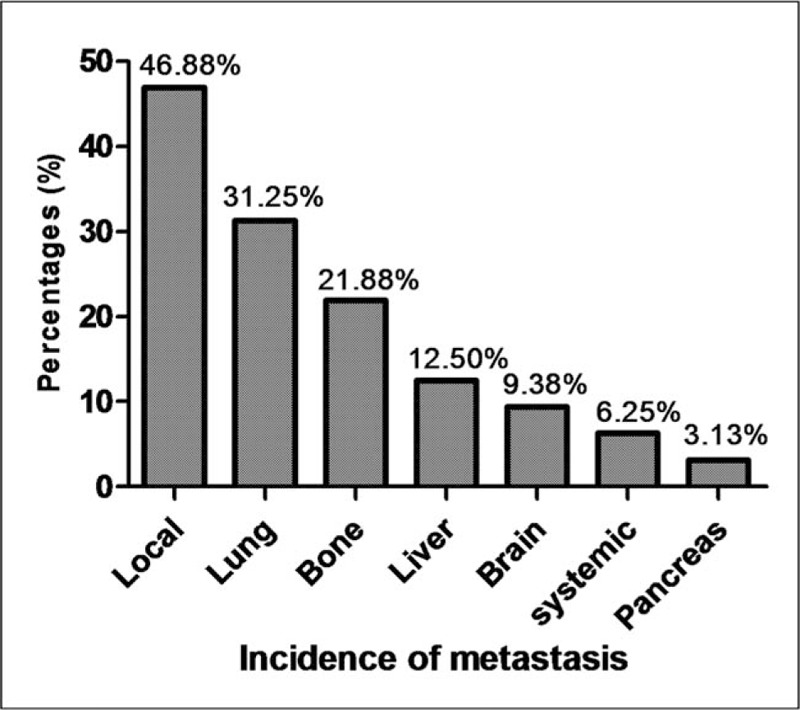
The incidence rate of metastases and the frequency of organ metastasis.

The metastasis of CBTs after surgery was confirmed in 32 cases (1.79%, Fig. 5), with most recurrences being local lymph node metastasis (15 cases). The locations of tumor cases of organ metastasis were the lung (10 cases), bone (7 cases), liver (4 cases), brain (3 cases), and pancreas (1 case). Systemic metastasis occurred in 2 cases (6.25%). Only 7 patients with recurrence underwent radiotherapy after surgery (21.88%), and no patients underwent chemotherapy. Death due to metastasis within 6 months was documented in 14 patients with tumor recurrence (43.75%).

## 4 Discussion

CBTs are very rare types of vascular tumors with a reported incidence of approximately 1:30,000.^[3]^ The incidence of CBTs in the Netherlands is reportedly 0.06 per 100,000.^[13]^ Most data on CBT management come from case reports. To date, there have been no large-scale case studies in Chinese patients. Therefore, we analyzed data from a decade of CBTs in China. The results demonstrated that the sex ratio of CBTs was 1:1.51 (male: female). The mean age at the first confirmed tumor was 40.6 years old, and the peak age of onset was during the 40- to 50-year-old age range. These findings are significantly different from those of a previous report that indicated there was no sex difference in incidence and that the average age was 34 years old for patients with CBTs.^[14]^ Our results showed that CBTs were more common in females than in males and that the peak age was older, which was consistent with other previous reviews.^[2,15]^

Some CBT patients show a familial tendency, with hereditary factors reflected in 1.49% of the cases in the present study, which was a lower incidence of familial CBTs compared to the 10% and 30% indicated in other reports.^[9,16]^ There was no striking predilection for CBTs on either side of the neck, and bilateral tumors were confirmed in 10% of sporadic cases^[17,18]^; however, in our study, the tumors appeared to be more common on the left side of the neck (right/left ratio was 1.26), and bilateral cases only accounted for 7.30% in Chinese patients. It has been previously corroborated that higher altitudes are related to the incidence of CBTs; in our study, 11.33% of patients came from high-altitude areas. Female patients from high altitudes more commonly had CBTs than patients from altitudes <1500 m above sea level (8.3:1)^[19]^; a possible reason for CBTs occurring more often in residents of high altitudes is because the atmospheric oxygen pressure is reduced and chronic hypoxia is produced, which induces hyperplasia of the carotid body.^[20]^

In our study, the CBTs had a mean volume of 33.4 cm^3^, which was greater than that reported in non-Asian patients (23.6 cm^3^).^[9,21]^ In our series, most patients received further examination after presenting with a painless neck mass, as has been reported by others.^[3,22]^ In China, the most common auxiliary examination was color Doppler ultrasound (87.29%), followed by computed tomography (79.06%), DSA (38.23%), and magnetic resonance imaging (19.50%). Color Doppler ultrasound is the first choice for the detection of CBTs because it provides noninvasive accurate detection of tumors with high sensitivity even when the tumors are not palpable and is cheaper for most Chinese patients. Modern CT or MR scans show infiltrations in the neck and provide definitive imaging for preoperative planning, including information about the tumor and data on vessel wall involvement.^[3,21,23]^ DSA is the gold standard for CBT detection and therapy because it can clearly display the details of the vascular structures adjacent to the tumor and can provide important information on blood flow and the patency of the circle of Willis. Therefore, DSA is essential for the assessment of CBTs in terms of the complex vascular anatomy and feeding vessels, especially for Shamblin type II and type III tumors.^[21,23,24]^ In our study, DSA was performed in only 38.25% of CBT patients in the past decade due to economic and technical reasons. Few patients could afford the cost of DSA; thus, only 8.6% of patients received preoperative embolization, according to our research.

The most common symptom in Chinese patients was similar to that in non-Asian patients, namely, a painless palpable neck mass (77.5%) located below the angle of the mandible that was not mobile vertically but was mobile laterally.^[25]^ In addition, 204 patients complained of a painful mass; other patients also showed symptoms of cerebral ischemia, TIA, cranial nerve compression, local occupation, etc.^[21,26]^ Previous reports demonstrated that parotid cysts, neurofibromas, carotid aneurysms, and other neck masses may be confused with CBTs, which may cause misdiagnosis. This error in clinical diagnosis has been reported in 30% to 40% of cases, and 20% of CBT patients are not diagnosed before their operation.^[27,28]^ In our study, 12 patients (0.7%) were not diagnosed before the operation and underwent a biopsy or inappropriate surgical resection. These data indicate that the rate of misdiagnosis in Chinese CBT patients was significantly less than that in previous reports, which might be related to China's medical policy and prior limited understanding of CBTs by Chinese doctors. For rare occurrences such as CBTs, performing a surgical procedure without an accurate diagnosis can be considered an illegal medical practice during the last few years in China.

Currently, surgical resection remains the gold standard for the treatment of CBTs. Traditional meticulous subadventitial resection has been the most common surgical approach to remove CBTs. This procedure, which commences at the bifurcation, may carry the risks of arterial injury and intraoperative bleeding;^[17,29]^ therefore, we recommended early control but not clamping of the common and external carotid arteries in all cases, which might be helpful for CBT resection. Shamblin type I tumors can be dissected in a capsular-adventitial plane without injury to vessels; however, as the tumor size increases and the type of CBT increases (i.e., types II and III), resection becomes more difficult with a higher incidence of complications.^[6,30]^ Thus, in most cases, vascular resection and reconstruction become inevitable. According to our results, some patients (26.12%) underwent complete tumor resection accompanied by vessel resection and reconstruction, with a saphenous vein, a graft, or an artery used for vessel repair. Another 26 patients underwent carotid ligation due to uncontrolled bleeding; however, carotid ligation is associated with a high risk of stroke and death and thus should be avoided.^[31]^ Moreover, patients with bilateral CBTs underwent staged resections (73.5%), which was the recommended procedure for bilateral cases because simultaneous excision might increase the risk of cranial nerve palsies, resulting in severe disabilities.^[32]^

The most common complication of CBT resection is cranial nerve injury, with an incidence ranging from 11% to 50%^[21,33–35]^; however, most nerve injuries are temporary, and recovery occurs within 6 months.^[3]^ In our study, the total incidence rate of nerve injury was 23.06%. The most commonly involved nerves were the hypoglossal nerve, vagus nerve, and sympathetic nerves (Fig. 3). Previous reports have shown that the incidence rate of nerve injury increases in tumor cases larger than 4 cm.^[36]^ Furthermore, the incidence rates of stroke and TIA after surgery range from 0% to 8%;^[37]^ different authors have reported different results, which can be related to the experience of the operating surgeons.^[3]^ In our study, stroke and TIA only occurred in 1.95% of the patients. Complex CBTs may lead to severe blood loss, stroke and mortality. Carotid shunting may play a vital role in the resection of CBTs and ensure blood supply to the brain during surgery, which decreases the risks of stroke and TIA.^[38]^ When the intraoperative internal carotid backpressure is <40 mm Hg, shunting should be used. In our research, we found that 3.9% of patients underwent surgical resection assisted by carotid shunting.

Several subsequent studies have demonstrated that preoperative selective embolization is an effective and safe method for surgical resection and helps to diminish intraoperative bleeding and malignancy.^[10,11]^ For this study, we collected relatively complete comparison data regarding embolization. Our results demonstrated that embolization could decrease intraoperative blood loss and shorten the procedure time, resulting in a significant clinical benefit for CBT therapy. Thus, many authors recommend preoperative embolization to diminish vascularity, thereby facilitating the meticulous dissection of large tumors. The potential complications associated with embolization are the migration of glue into the intracranial circulation, chemical toxicity, and direct puncture.^[39]^ The major risk of stroke from embolization was reported to be as high as 17% in a single-center study,^[6]^ and other authors have suggested that embolization provides little benefit in CBT management. Although recent review demonstrated that preoperative embolization could reduce blood loss and complications as well as improves tumor excision,^[15]^ this technique should be used strictly by experienced surgical teams and should not be used indiscriminately in all cases.

The incidence rate of malignant CBTs has been reported to be ∼5% to 10%, and most CBT cases involve local malignancy or regional lymph node metastasis, while systemic metastasis is rare, according to previous reports.^[6,7,40]^ In our study, 79.2% of metastases were confirmed via pathology. These cases displayed the histologic features described by Lack, namely, central necrosis of the clusters, invasion of the vascular spaces, and mitoses.^[41]^ However, other authors believe that the presence of metastases in lymph nodes or distant organs is the only acceptable evidence of malignancy^[6,7,40]^; therefore, 16 patients (20.8%) were confirmed as having a malignancy, according to this feature. The incidence of metastasis (1.79%) was lower than that reported in Western patients.^[6,7]^ The most frequent metastatic sites were local (46.9%), lung (31.3%), bone (21.9%), liver (12.5%), and brain (9.4%). Our results depict the metastatic characteristics of CBTs in a Chinese cohort, as these had not previously been compiled. As CBTs are rare tumors, long-term control of CBTs was obtained in 94% of patients who underwent surgical treatment.^[42]^ In our study, the overall survival rate was 98.87% at 36 months and 56.2% at 6 months for patients with tumors that had metastasized. Radiotherapy was proven effective in a few cases, and CBTs are not sensitive to chemotherapy; therefore, radiotherapy alone was recommended for giant and recurrent tumors as well as for patients who refused surgical treatment.^[43]^ In the present study, only 21.9% of patients with metastases underwent radiotherapy, and no patients in the last decade underwent chemotherapy. A recent paper described that fractionated external beam radiotherapy produced a better outcome than conventional radiotherapy in CBT patients.^[42]^

## 5 Conclusions

This was the first large-scale study to describe the clinical features of CBTs in Chinese patients in the last decade; these features are listed as follows:

1. Females accounted for 60.2% of cases, with a peak age of 40 to 50 years old.
2. Most CBTs were sporadic (98.5%), and bilateral cases accounted for only 7.3%.
3. The most common presentation was a painless neck mass (77.5%), and the rate of misdiagnosis was 0.7% in all cases.
4. Surgical resection was the gold-standard treatment, with 26.63% of patients undergoing partial vessel resection. Vessel replacement materials included veins (68.7%), grafts (19.3%), and the external carotid artery (12.0%).
5. Selective embolization was only used in a small number of cases. This technique could significantly decrease the procedure time and intraoperative blood loss.
6. Most cranial nerve injuries were temporary, with an incidence rate of 23.1%. Common presentations were tongue slant (7%) and hoarseness (6.1%).
7. The malignancy rate was 4.3%, and the overall survival rate was 98.87% at 36 months. Local metastases accounted for 46.7%, and the locations of organ metastasis were the lung (31.3%), bone (21.9%), liver (12.5%), and brain (9.4%).
8. Only 21.9% of metastatic patients underwent radiotherapy, and the survival rate was 56.2% at 6 months. No patients underwent chemotherapy.

## Acknowledgments

The language in this article was edited by American Journal Experts (AJE).

## Author contributions

**Conceptualization:** Yanzi Li, Jianlin Liu, Lin Yang.

**Data curation:** Yangjing Chen, Yanzi Li, Jianlin Liu, Lin Yang.

**Formal analysis:** Yangjing Chen, Yanzi Li, Jianlin Liu, Lin Yang.

**Investigation:** Yangjing Chen, Yanzi Li, Jianlin Liu, Lin Yang.

**Methodology:** Yangjing Chen, Yanzi Li, Lin Yang.

**Project administration:** Jianlin Liu, Lin Yang.

**Resources:** Yangjing Chen, Yanzi Li, Lin Yang.

**Software:** Yangjing Chen, Yanzi Li, Jianlin Liu, Lin Yang.

**Supervision:** Yanzi Li, Jianlin Liu, Lin Yang.

**Validation:** Yangjing Chen, Lin Yang.

**Visualization:** Yangjing Chen, Lin Yang.

**Writing – original draft:** Yangjing Chen, Yanzi Li, Lin Yang.

**Writing – review & editing:** Jianlin Liu, Lin Yang.
